# Dietary Supplementation on Physical Performance and Recovery in Active-Duty Military Personnel: A Systematic Review of Randomized and Quasi-Experimental Controlled Trials

**DOI:** 10.3390/nu16162746

**Published:** 2024-08-17

**Authors:** Jacie Harlow, Kylie Blodgett, Jenna Stedman, Rachele Pojednic

**Affiliations:** 1 Department of Health and Human Performance, Norwich University, Northfield, VT 05663, USAkcowens@norwich.edu (K.B.); 2 Department of Nutrition & Dietetics, Kansas University Medical Center, Kansas City, KS 66103, USA; jstedman@kumc.edu; 3 Stanford Lifestyle Medicine, Stanford Prevention Research Center, Department of Medicine, Stanford University, Stanford, CA 94305, USA

**Keywords:** warfighter, supplementation, muscle, performance, recovery

## Abstract

Background: Warfighters, often called tactical athletes, seek dietary supplementation to enhance training and recovery. Roughly 69% of active-duty US military personnel have reported consuming dietary supplements. The objective of this systematic review was to examine the impact of dietary supplements on muscle-related physical performance and recovery in active-duty military personnel. Methods: Randomized controlled trials and quasi-experimental controlled trials of oral dietary supplementation in active-duty military members were examined. A protocol was registered (PROSPERO CRD42023401472), and a systematic search of MEDLINE and CINAHL was undertaken. Inclusion criteria consisted of studies published between 1990–2023 with outcomes of muscle performance and recovery among active-duty military populations. The risk of bias was assessed with the McMaster University Guidelines and Critical Review Form for Quantitative Studies. Results: Sixteen studies were included. Four were conducted on protein or carbohydrate; four on beta-alanine alone, creatine alone, or in combination; two on mixed nutritional supplements; two on probiotics alone or in combination with beta hydroxy-beta methylbutyrate calcium; and four on phytonutrient extracts including oregano, beetroot juice, quercetin, and resveratrol. Ten examined outcomes related to physical performance, and six on outcomes of injury or recovery. Overall, protein, carbohydrate, beta-alanine, creatine, and beetroot juice modestly improved performance, while quercetin did not. Protein, carbohydrates, beta-alanine, probiotics, and oregano reduced markers of inflammation, while resveratrol did not. Conclusions: Nutrition supplementation may have small benefits on muscle performance and recovery in warfighters. However, there are significant limitations in interpretation due to the largely inconsistent evidence of ingredients and comparable outcomes. Thus, there is inadequate practical evidence to suggest how dietary supplementation may affect field performance.

## 1. Introduction

Tactical athletes form a unique cohort of professionals, encompassing military personnel and warfighters, firefighters, law enforcement officers, and first responders who face the dual occupational demands of rigorous physical requirements and potential exposure to life-threatening scenarios [1]. These individuals are tasked with meeting stringent physical criteria and job-specific expectations that necessitate optimal health and performance. While the training regimens of military personnel, specifically, equip them to endure substantial challenges, the exigencies of sustained exertion can eventually impede performance and overall well-being. As such, military personnel will often seek nutritional support and supplementation to enhance training and recovery [2,3]. Indeed, the majority of military personnel have reported dietary supplement use, with those in combat-related occupations reporting the highest use in a recent survey of all US Armed Forces personnel [4]. According to the US Army, soldiers reported consuming supplements to promote general health, muscle strength, and overall performance [5]. Altogether, these reports suggest there is an underlying assumption among military personnel that supplementation is an effective means for improving physical performance and recovery outcomes.

This is further supported by recent data demonstrating the utilization of dietary supplements is greater in the military than in civilian populations [4,5,6]. In a large survey of service members, 74% of all military personnel reported using more than one dietary supplement per week, with 40% utilizing more than five per week [5]. Multivitamins/multiminerals were the most commonly used supplement, followed by combination products, proteins (amino acids), individual vitamins/minerals, herbals, joint health products, and purported prohormones [5]. Interestingly, females, those over age 40 years, and those with a higher education level and rank were more likely to report greater supplement use [5]. Similar unique demographic stratifications were also noted in a prior analysis extracted from the 2011 Department of Defense Survey of Health-Related Behaviors [4]. The utilization of dietary supplements by soldiers also appears to increase over time during their years of service [3].

While supplementation use is high in the military population, there are limited investigations assessing the effects of dietary supplementation on physical performance in tactical athletes or military personnel. There is, however, a larger body of work addressing the effects of supplementation in sports athletes [7]. Unfortunately, sports performance outcomes have little generalizability to the tactical population. Military personnel spend extended periods of time in field training, often in extreme weather conditions and with extensive personal protective equipment. Requirements to maximize performance and recovery in this environment are unique. Moreover, the limited data on the benefits of dietary supplementation on field outcomes in the tactical population, specifically outcomes of performance and recovery, are mostly self-reported or cross-sectional in nature [2,3,4,5].

There is a critical need to understand the efficacy, dosing, and safety of dietary supplements in military and tactical populations, as muscle performance and recovery outcomes are paramount for warfighters, especially when deployed or in the field where these populations are often required to perform without reliable access to a well-balanced diet. Despite the unique occupational stress and repeated performance requirements, there is still limited data to understand the outcomes of supplementation in these tactical athlete populations. While the appropriate use of some supplements may benefit military personnel, others may cause harm to health, performance, career, and reputation. This is particularly salient, given a recent report that demonstrated the majority of weight loss supplements marketed to members of the military are labeled inaccurately, with some in direct violation of the Department of Defense Prohibited Dietary Supplements Ingredients List (www.opss.org) [8]. The purpose of this systematic review is to investigate the effect of dietary supplementation on physical performance and recovery in active-duty military personnel in randomized and quasi-experimental controlled trials to elucidate potential benefits or harms to warfighters.

## 2. Materials and Methods

A systematic review was registered with the international prospective register of systematic reviews (PROSPERO CRD42023401472) and all methods complied with the Preferred Reporting Items for Systematic Review and Meta Analysis Protocols (PRISMA-P). Medline and CINAHL were used to find studies addressing the effects of dietary supplementation on physical performance and recovery in military populations. Search terms are summarized in Table 1.

Criteria used to define study inclusion are summarized in Table 2. Studies were included if they were randomized or quasi-experimental controlled trials published in the English language between 1990–2023, with outcomes on physical performance and recovery among active-duty military populations (ages 19+). Studies were excluded if the design was case-control, cohort, cross-sectional, or meta-analysis, if the population of focus was retired military personnel, or if the study did not measure physical outcomes related to muscle or use supplements approved by the Department of Defense (DoD).

**Figure 1:**
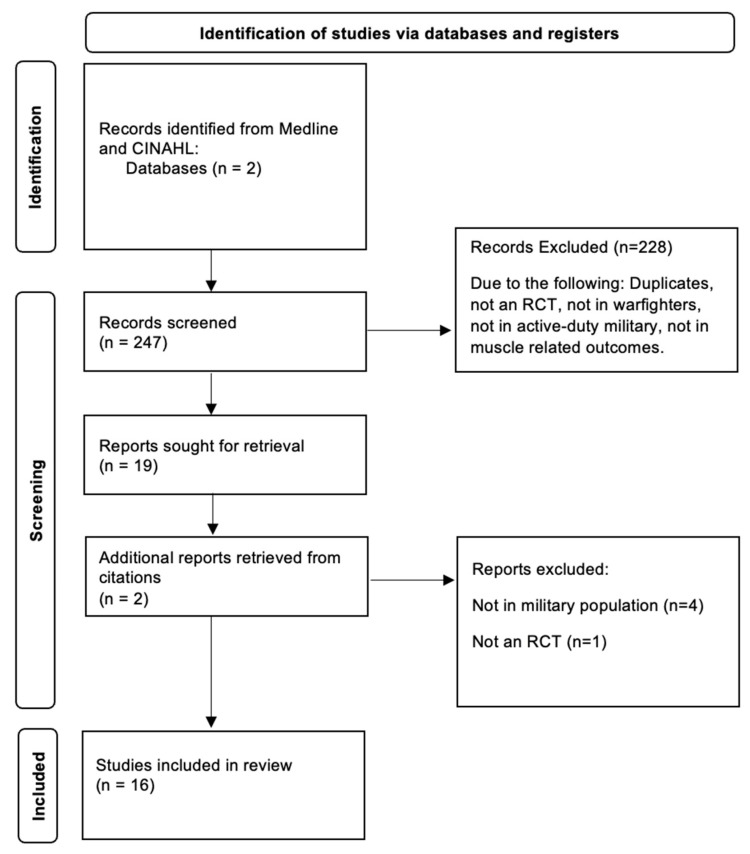
Preferred reporting items for systematic review and meta-analysis protocols (PRISMA-P) selection of studies.

Abstracts from each search were uploaded into Rayyan (rayyan.ai) [9], a free web application designed to facilitate the screening process for duplicate deletion and title/abstract review. In order to increase inter-rater reliability, upon completion of the abstract review, two members of the research team assessed the full text of the remaining manuscripts for final eligibility. If there were any disagreements about inclusion, a third member of the research team was consulted, and consensus was reached. A risk of bias assessment was performed, and only articles that were deemed to be of acceptable quality according to McMaster University Guidelines and Critical Review Form for Quantitative Studies [10] were included. Any article that did not meet all inclusion criteria was excluded (Figure 1).

Data from the selected articles were copied to a Microsoft Excel (Version 16.75.2) spreadsheet by one author (JH) and checked by a second author (RP). Disagreements were resolved by reaching a consensus. The following information was recorded: author’s name, publication year, study country, number of participants, mean age, intervention, method of exposure assessment, outcome results, and methodological quality score (Table 3).

## 3. Results

The initial search yielded 247 articles, 228 of which were excluded based on criteria outlined in Figure 1. Two additional reports were retrieved based on citations of retrieved studies. Sixteen (n = 16) manuscripts were included that examined the relationship between supplementation and physical performance OR recovery and injury in active duty military populations. Four (n = 4) examined protein or carbohydrate, in combination or alone [12,20,21,25]. Four investigated (n = 4) beta-alanine alone [15,16], creatine alone [11], or beta-alanine and creatine in combination [22]. Two (n = 2) examined mixed nutritional supplements [13,26]. Two (n = 2) investigated probiotics (bacillus coagulans) alone [17] or in combination with beta hydroxy-beta methylbutyrate calcium (CaHMB) [14]. Four (n = 4) examined phytonutrient extracts, including oregano [24], beetroot juice [19], quercetin [23], and resveratrol [18].

### 3.1. Protein and Carbohydrate

Four studies were conducted on protein supplementation, including whey protein in combination with casein or leucine and whey protein in comparison with carbohydrate supplementation. Whey plus casein had similar body composition and performance outcomes to carbohydrate supplementation in a refeeding trial post severe energy restriction [12], whereas whey plus leucine was associated with increases in fat-free mass (FFM) and strength performance [25]. Whey supplementation caused improvements in overall body composition compared to carbohydrate supplementation, without differences in strength performance [20]. Whey supplementation also attenuated the musculoskeletal injury rate and decreased limited duty days compared to no supplementation but had similar outcomes to carbohydrate supplementation [21].

Berryman and colleagues [12] investigated the effects of high-quality protein supplementation on (FFM) restoration after a short-term (7 days) severe negative energy balance caused by Survival Evasion, Resistance, Escape (SERE) school. Sixty-three (n = 63) US Marines received either high protein (1.6 g/kg), moderate protein (1.0 g/kg), or low protein (0.1 g/kg) supplementation in addition to an ad libitum diet for 27 days. High and moderate protein groups received two whey protein supplements and one casein supplement each day, while the low protein group received a flavor-matched carbohydrate beverage. Overall, participants lost 7.0% total body mass and 4.7% FFM during SERE school. However, body composition measures returned to baseline after 27 days of intervention in all groups, with no significant difference between groups. Interestingly, all Marine participants met or exceeded current recommendations for protein, and authors hypothesized that in the presence of adequate protein, restoration of caloric intake rather than higher levels of protein was responsible for driving FFM recovery. The study team measured body composition via dual-energy X-ray absorptiometry (DEXA), which is inherently limited during severe caloric restriction. Additionally, the study did not measure functional muscle outcomes to understand the implications for functional recovery.

A whey protein plus leucine supplement was examined by Walker and colleagues [25] to understand the potential enhancement of overall cognitive and physical performance in active-duty Air Force males in a double-blind, randomized controlled trial (RCT) that lasted eight weeks. The protein supplement group (n = 18) received a 112 kcal packet with 19.7 g of whey protein and 6.2 g leucine per day, and the placebo supplement group (n = 12) received 112 kcal of carbohydrate with 0.0 g of protein once daily for eight weeks. Participants’ body composition, cognition, and physical fitness were assessed at baseline and eight weeks. Post-intervention, bench press, pushups, total mass, fat-free mass, and lean body mass all increased in the protein supplement group (*p* < 0.05) but not in the placebo group or between groups. No differences were observed within or between groups for crunches, chin-ups, or 3-mile run time. No significant cognitive performance outcomes were observed. Limitations of this study included a short supplementation time frame of 8 weeks, no diversity in gender, and a lack of control over diet resistance training or physical training behaviors.

To understand whether whey protein supplementation alone compared to carbohydrate supplementation alone can improve strength performance among military populations, McAdam et al. [20] conducted a double-blind, parallel-group RCT on United States soldiers during Army Initial Entry Training (n = 69). Two platoons of soldiers received two daily servings of whey protein (77 g/day), while another two platoons received two daily servings of a carbohydrate supplement (127 g/day) matched in calories and overall taste for eight weeks. Body composition was assessed during weeks 1 and 9, and physical performance was evaluated by the Army Physical Fitness Test (APFT) during weeks 2 and 8 of the training. Results demonstrated an overall reduction in FFM pre-training versus FFM post-training (F = 4.63, *p* = 0.04) and a significant group difference in fat mass (kg) reduction between groups (*p* < 0.05), with the whey protein group losing an additional 1.8 kg of fat mass. While all platoons improved scores in pushups, sit-ups, and run time, there was no significant difference between supplement groups in any test outcome. The limitations of the study include the use of skinfold measurements, which can be a less reliable modality of assessing body composition, and the variance among different platoons due to different training instructors.

To examine the effect of protein and carbohydrate supplementation on injury and limited duty, McGinnis and colleagues [21] compared carbohydrate to protein supplementation during Army Initial Entry Training. A double-blind RCT was conducted on four groups of Initial Entry Training soldiers that consumed either (1) one whey protein (38.6 g, 293 kcal); (2) one carbohydrate (63.4 g, 291 kcal); (3) two whey protein (77.2 g, 586 kcal); or (4) two carbohydrate servings/day (126.8 g, 582 kcal) after physical training and before bed, or before bed only for 15 weeks. Injury data rates, musculoskeletal injury (MSI) rates, and limited duty rates were compared across supplement groups and with historical non-supplemented controls. There were no significant differences in injury rate or limited or missed duty rates between protein and carbohydrate supplement groups or between one supplement per day of either kind and no supplement. When carbohydrate and protein supplement groups were combined, soldiers who did not receive any supplementation had a 5.35-fold elevated risk for injury when compared to soldiers who supplemented twice per day (*p* = 0.002). Additionally, soldiers who consumed two servings per day were also less likely to be injured than those consuming one serving, regardless of supplement type (*p* = 0.002). Limitations of this study included lack of diversity in gender, comparison to historical data of non-supplemented soldiers, and the lack of oversight of supplementation as supplements were distributed by drill sergeants rather than study personnel, nor was daily diet controlled. 

### 3.2. Beta-Alanine and Creatine

Four studies were conducted on beta-alanine alone [15,16], creatine, or beta-alanine and creatine in combination [22]. Beta-alanine alone appeared to improve occupational-related testing [15] and reduce inflammation after intense military training [16]. Beta-alanine and creatine, in combination, improved lab- and field-based performance tests, although they did not demonstrate improvements in muscle-related hormones or growth factors [22]. Short-term creatine supplementation alone demonstrated no improvements in exercise performance, body composition, or health-related metrics [11].

In order to determine if beta-alanine supplementation can influence muscle carnosine levels among soldiers, a double-blind RCT examined beta-alanine (6 g per day) versus placebo (6 g rice flour) in male Israel Defense Force Soldiers (n = 18) over a 30-day period [15]. Soldiers completed various military performance tests to assess cognitive and physical performance at baseline and post-intervention. Carnosine content of the gastrocnemius and brain were assessed utilizing magnetic resonance spectroscopy (MRS). Post-intervention, carnosine levels within the gastrocnemius were higher in the beta-alanine group than the placebo (*p* = 0.048) and moderately correlated to changes in the rate of fatigue during the 1-min sprint (r = 0.63, *p* = 0.06), although this relationship did not reach statistical significance. No notable differences were observed after the intervention in either group in brain carnosine content, marksmanship, or performance in the 2.5 km run, 1-min sprint, and 30 s repeated sprint. Participants in the beta-alanine group did significantly improve their time for the 50-m casualty carry (*p* = 0.044) and increased their performance in the Serial Sevens subtraction test compared to placebo (*p* = 0.022), suggesting some physical and cognitive benefit, despite a lack of brain carnosine changes. Limitations included a small sample size, a potential lack of compliance with supplementation intake, and no control for daily nutritional intake.

Beta-alanine supplementation was also examined among military personnel for recovery after intense training and exercise. Hoffman and colleagues [16] conducted a double-blind RCT on male Israel Defense Force Soldiers (n = 20) comparing beta-alanine supplementation (12 g per day) to placebo for seven days between two intensive periods of navigational training and restricted sleep. Circulating cytokine Interleukin 10 (IL-10) concentrations were measured after the first period of navigational training before supplementation began and then again after the seven-day supplement regimen and second bout of training. Results indicated that the change in circulating IL-10 concentrations from pre to post-supplementation (mean difference 0.86 pg/mL) was possibly greater for beta-alanine than placebo using a qualitative mechanistic inference based on *t*-tests. The limitations of this study include no diversity in gender, small sample size, and lack of performance measures.

Samadi and colleagues [22] investigated the combination of beta-alanine and creatine in a double-blind RCT that included male soldiers between 19 and 25 years old (n = 20). All participants were supplemented with 6.4 g/day of beta-alanine for 28 days. After the third week, all participants continued with beta-alanine and were split to receive either additional creatine (0.3 g/kg/day) or isocaloric placebo control for seven days. Outcomes included physical fitness testing procedures: running anaerobic sprint test (RAST), 1 rep max (1RM) for leg press and bench press, the vertical jump test, and the Simulated Casualty Evacuation Test (SCET). Blood samples were taken to analyze testosterone and cortisol levels. Results showed significant increases in the beta-alanine plus creatine group in the bench press (*p* = 0.026), leg press (*p* = 0.017), vertical jump (*p* = 0.009), as well as a decrease in time for the SCET (*p* = 0.003). There were also significant increases in testosterone (*p* = 0.006) with both beta-alanine plus creatine (5.46 ± 0.66 vs. 5.92 ± 0.65), as well as beta-alanine plus placebo (5.61 ± 0.59 vs. 5.64 ± 0.64). There were no significant changes in cortisol (*p* = 0.551), IGF-1 (*p* = 0.116) or lactate (*p* = 0.090) within or between groups. The limitations of this study include limited blood sampling post-exercise, a lack of creatine content measurement in muscle, a lack of diversity in gender, and no true placebo or creatine-only group.

A double-blind RCT that included thirty-five (n = 35) United States Soldiers (n = 20 men) was conducted to determine if creatine supplementation alone increased endurance performance [11]. Participants were assigned to consume either 20 g of creatine (5 g four times per day) or 20 g of taurine (5 g four times per day) for seven days. Outcomes consisted of 2 min of pushups, diastolic blood pressure, serum creatine phosphokinase levels, and serum creatinine concentration before and after supplementation. Results show no evidence of significant differences between the creatine and taurine groups for pushup performance, blood pressure, body composition, weight, or serum creatine phosphokinase. However, the creatine group did demonstrate an increase in serum creatinine compared to placebo (*p* < 0.001). The limitations of this study included the small sample size and short duration of supplementation.

### 3.3. Mixed Nutritional Supplements

Two studies examined the effects of mixed nutritional supplements [13,26] on physical performance outcomes. One demonstrated improvements in fitness-related exercise tests as well as cognition [26] after a multimodal training and novel nutrition supplement intervention. One demonstrated attenuated losses of overall body mass, lean mass, and physical performance after intense military training, with no effect on circulating growth factors or hormones [13].

Zwilling and colleagues [26] examined mixed nutritional supplements in a 12-week RCT that included men and women in the Air Force. A total of 148 (28% female) Airmen were split into two groups: exercise and supplement (n = 70) or exercise and placebo (n = 78). The supplement was reported as a 267 kcal 8-ounce drink that included protein, β-hydroxy β-methyl butyrate (HMB), lutein, phospholipids, DHA, Vitamin D, and multiple micronutrients. The placebo was a 100-kcal drink that contained only protein (2.0 g) and carbohydrate (20.0 g). All Airmen participated in resistance training, endurance, and high-intensity total body work five times per week for at least 45 min and consumed supplements or a placebo two times per day for 12 weeks. All outcomes were tested pre- and post-intervention and included changes in physical fitness (power, strength and endurance, mobility and stability, blood pressure, heart rate, and lean muscle mass), cognitive assessment (short-term memory, episodic memory, fluid intelligence, working memory, executive function, and processing efficiency and reaction time), and plasma biomarkers (B12, folate, triglycerides, cholesterol, ferritin, cortisol, fatty acids, and lutein). Results indicated that 83% of the metrics post-intervention changed. There were significant increases in power (*p* = 0.001), strength and endurance (*p* < 0.001), mobility and stability (*p* < 0.001), heart rate (*p* = 0.018), and lean muscle mass (*p* = 0.001) within and between groups. Supplementation increased plasma vitamin B12, folate, DHA, and lutein. Additionally, the exercise training plus placebo intervention improved eight individual measures of cognition, while the exercise training plus nutritional supplement intervention effectively improved 11 individual measures of cognition. Limitations of the study included not controlling for baseline concentration of biomarkers and lack of a control group.

Fortes and colleagues [13] examined a mixed nutritional supplement compared to control (no supplement) on body composition via DEXA, physical performance (maximal dynamic lift strength, vertical jump, and explosive leg power), and circulating serum markers (IGF-1, IGF BP-1, IGF BP3, testosterone, and cortisol) in male soldiers (n = 30) before and after eight weeks of military training. The supplement provided an additional 5.1 MJ/day of energy in a bar consisting of 45% carbohydrate, 40% fat, and 15% protein. The supplement group experienced attenuated losses of overall body mass, lean mass, and physical performance compared to control. There were no significant differences between groups in terms of circulating growth factors or hormones, except the loss of IGF-BP3 was attenuated in the supplement group. Limitations of the study included a small sample size, a male-only population, and a short supplementation time period.

### 3.4. Probiotics

Two studies were conducted on probiotics (*Bacillus coagulans*) [14,17]. Probiotics alone demonstrate no change in physical performance outcomes [17]. Probiotics in combination with beta hydroxy-beta methyl butyrate calcium (CaHMB) demonstrated positive effects on muscle integrity via MRI and improvements in circulating markers of muscle metabolism and cytokines after military training [14].

To determine whether *Bacillus coagulans* probiotic supplementation has the potential to reduce inflammation in military personnel, a double-blind, placebo-controlled RCT was conducted on Israel Defense Force Soldiers (n = 15) [17]. The supplementation protocol consisted of two weeks of inactivated *B. coagulans* (1.0 × 10^9^ colony units) or a placebo dissolved into water once per day. Physical exercise was consistent throughout the 14-day duration, and soldiers conditioned together five days of the week. The pre- and post-physical assessments included vertical jump power, 60 s maximum pull-ups, a casualty drag, and a 2 × 100 m shuttle run. Resting blood measures for testosterone, cortisol, creatine kinase, and inflammatory cytokines were also assessed. Pre- and post-scores for physical performance outcomes and biomarkers indicated no change, although trends in the probiotic groups indicated potential benefit compared to placebo. Limitations of this study include the inability of some soldiers to complete the POST testing protocol, the small sample size, and a lack of daily dietary control.

Gepnar et al. [14] conducted a double-blind, parallel RCT that analyzed inflammatory response and muscle integrity of male soldiers of the Israel Defense Force (n = 25) after 40 days of intense training. Participants were randomized to one of three groups: *B. coagulans* (BC30) with Beta hydroxy-beta methyl butyrate calcium (CaHMBBC30), CaHMBPL alone, or placebo. The 40-day long training period consisted of hand-to-hand combat simulation, garrison/classroom environment, and navigation through rigorous terrain. Blood samples were taken to analyze serum creatine kinase, lactate dehydrogenase, and cytokines, and magnetic resonance imaging was completed before the 40-day period started and 12 h after all physical events on the final day of training. Significant attenuations were noted in IL-1, IL-2, IL-6, CX3CL1, and TNF- for both CaHMBBC30 and CaHMBPL compared with control. Plasma IL-10 concentrations were significantly attenuated for CaHMBBC30 compared with control only. Additionally, there were positive effects on muscle integrity via MRI in the rectus femoris muscle and vastus lateralis when the results collapsed across all the supplement groups compared to placebo. Limitations of this study were the lack of body composition and performance measures, a lack of dietary control, and no diversity in gender.

### 3.5. Phytonutrients

Four studies were conducted on phytonutrient extracts [18,19,23,24]. Oregano extract supplementation indicated a potential acute beneficial effect on markers of muscle damage and oxidative stress [24]. Beetroot juice appeared to attenuate declines in fitness and heart rate recovery after a field expedition [19]. Quercetin did not improve physical performance metrics or perceived exertion during exercise testing [23]. Resveratrol did not appear to improve serum markers of muscle metabolism or antioxidant enzyme activity [18].

To understand how antioxidant supplementation affects the inflammatory response to high-intensity training in soldiers, Shirvani et al. [24] investigated the effects of oregano supplementation on muscle damage, oxidative stress, and plasma antioxidant markers of soldiers performing the army combat readiness test (ACRT). Twenty-four healthy male soldiers (age: 24 ± 3 years) were randomized to receive a placebo (n = 12) or 500 mg of an oregano supplement (n = 12) immediately post-ACRT. ACRT increased markers of muscle damage and oxidative stress in all participants. Plasma creatine kinase (CK), lactate dehydrogenase (LDH), malondialdehyde (MDA), superoxide dismutase (SOD), glutathione peroxidase (GPX) and total antioxidant capacity (TAC) were pre-, immediately post-, 60 min and 120 min post-ACRT. Oregano supplementation resulted in decreased CK, LDH, and MDA (*p* < 0.0001) and increased SOD, GPX, and TAC (*p* < 0.0001) capacity at all time points. Results indicate a potential acute beneficial effect on markers of muscle damage and oxidative stress after oregano supplementation. Limitations of the study included a short measurement time period post-ACRT and a small sample size with a lack of gender diversity.

Beetroot juice (BRJ) can provide dietary nitrate (NO3^−^), which could potentially improve performance by inducing endogenous nitric oxide (NO) synthesis. Marshall and colleagues [19] investigated the effect of BRJ compared to placebo on salivary nitrate (a potential precursor of NO), exercise performance, and high altitude acclimatization in twenty-two healthy adult participants (n = 12 men) taking part in the British RAF 100 Himalayan Venture 18 expedition. Participants were supplemented with two 70 mL per day of BRJ (~12.5 mmol nitrate per day) or non-nitrate calorie-matched control for three days prior to departure through the final day (day 17) of the expedition. Those supplemented with two 70 mL doses of BRJ experienced increased concentrations of salivary nitrate (*p* = 0.007) compared to baseline. In those who received a placebo, fitness scores declined (*p* = 0.003), and heart rate recovery was prolonged (*p* < 0.01) after the ascent, and these metrics were not affected in those receiving BRJ. Limitations of the study included fitness index scores rather than VO2max or performance outcomes, limited control on supplement dosage, and use of saliva nitrate measurements rather than urine.

To determine if quercetin supplementation can increase soldiers’ performance, Sharp et al. [23] conducted a placebo-controlled, cross-over, double-blind RCT with soldiers (n = 16) consuming quercetin bars (500 mg quercetin each) or an indistinguishable placebo twice per day for 8.5 days each. Participants’ body composition, Vo2peak, loaded treadmill marching, and cycling time trials were completed before and after the 8.5-day trial under each condition. Oxygen consumption, heart rate, and rate of perceived exertion were recorded during each exercise test. Results showed that plasma quercetin levels did not vary with the placebo group but did show elevated levels in the quercetin supplement group (239.2 ± 4.8 ng·mL, *p* < 0.001). However, supplementation with quercetin did not improve aerobic capacity, aerobic performance, steady state load carriage exercise, or change the metabolic or perceptual responses to exercise as compared with placebo. The study was limited by a lack of diverse subject gender and age, a small sample size, and a short time period of supplementation. 

Macedo and colleagues [18] investigated the effects of 90 days of 100 mg of resveratrol daily (n = 30), as compared to placebo (n = 30), on markers of metabolism and indicators of oxidative stress in military firefighters from Soldiers Training Course in the School for Military Police and Firefighters in Brazil before and after a standardized fitness test. Supplementation with resveratrol did not affect biochemical parameters (cholesterol, creatine kinase, lactate dehydrogenase, serum iron, hepatic enzymes, or creatinine), although glucose and triglycerides were elevated post-test after resveratrol supplementation (*p* < 0.05). Total antioxidant capacity, as determined by the ferric-reducing ability of plasma (FRAP assay), was increased in both groups after the fitness test but was not moderated by resveratrol supplementation. Antioxidant enzyme activity (SOD, catalase, Gpx glutathione reductase) was not affected by supplementation. However, GPx was significantly reduced (*p* < 0.05) post-FT in those taking resveratrol. Markers of oxidative damage (thiol groups, 8-isoprostane, and 8OHdG) were not different between groups before or after the FT. Of the pro-inflammatory cytokines that were measured (IL-6, IL1b, and TNFa), IL-6 and TNFa were reduced by 29% and 39% after the FT in those who received resveratrol compared to placebo. The authors believe the fitness test was not sufficiently intense enough to induce a response of free radicals and that the outcomes were limited in interpretation and generalizability.

## 4. Discussion

To our knowledge, this is the first systematic review to examine randomized or quasi-experimental controlled trials of dietary supplementation, specifically on physical performance and recovery metrics in the warfighter population. This topic is of critical importance as operational effectiveness and safety often hinge on the ability to perform and recover quickly in the field, a process that could be augmented with dietary supplementation. We identified sixteen (n = 16) intervention studies published between 1990–2023. Supplementation strategies varied across the study protocols, as did dose and length of intervention. Nine studies [11,13,17,19,20,22,23,25,26] examined physical and exercise test performance outcomes. Seven studies [12,14,15,16,18,21,24] investigated metrics associated with injury or recovery. No studies reported adverse events or harm. These results are potentially helpful for warfighter decision-making regarding personal dietary supplementation choices or healthcare providers like primary care physicians or dietitians who may be asked to make recommendations for supplementation. Importantly, these results highlight a critical gap in the warfighter supplementation literature that is paramount to readiness, performance, and recovery in the field.

### 4.1. Supplement Use for Muscle-Related Physical Performance and Recovery

Although efficacy data on warfighter dietary supplementation are lacking, soldiers report outcomes such as promotion of general health, increases in muscle strength, and enhanced performance [5]. Even among sports athletes, where evidence is more robust, there are limited conclusions to suggest definitive muscle-related improvements [7]. In both populations, it is unknown whether increases in performance or recovery are because supplements help to correct deficiencies due to dietary inadequacy or indeed provide unique physiologic benefits [7]. However, there are some plausible mechanisms.

Regarding muscle-related physical performance, dietary supplementation may play a key supportive role, particularly through the strategic use of targeted energy intake (i.e., carbohydrates or creatine) or through ingestion of singular micronutrients that could play a cofactor role in the regulatory processes that enhance muscle protein synthesis (i.e., protein) or cellular function (i.e., beta-alanine or polyphenols). For example, in sports athletes, it has been noted that carbohydrates are critical for maintaining glycogen stores, which fuel prolonged and high-intensity exercise. Adequate carbohydrate intake ensures that muscles have a readily available energy source, preventing premature fatigue and sustaining performance during extended physical exertion [7]. Creatine supplementation enhances intramuscular phosphocreatine levels, which are crucial for the rapid regeneration of ATP during short bursts of high-intensity exercise. This improved ATP availability translates to greater power output, strength, and overall performance in activities requiring quick, explosive movements [27]. Protein is essential for providing the amino acids required for muscle protein synthesis, which supports muscle hypertrophy and strength adaptations. When consumed pre- or post-exercise, protein helps optimize muscle protein turnover, thereby enhancing performance over time [28,29]. Beta-alanine serves as a precursor to carnosine, a dipeptide that buffers intramuscular hydrogen ions. By mitigating the accumulation of these ions, beta-alanine delays the onset of acidosis and muscle fatigue during high-intensity, anaerobic activities, thus extending the capacity for sustained effort [30].

**Figure 2:**
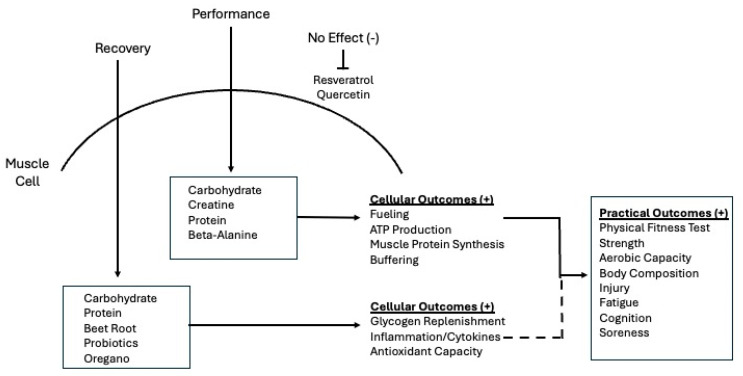
Proposed model for the effects of dietary supplementation on warfighter muscle-related performance and recovery, as well as practical field-based outcomes. Based on n = 16 experimental and quasi-experimental studies reviewed, evidence indicates that supplementing with carbohydrates, protein, beetroot juice, probiotics, and oregano may have an indirect effect on recovery by modulating fuel stores, inflammation, and repair. Carbohydrates, protein, creatine, and beta-alanine have a direct impact on acute muscle performance by influencing cellular processes that are necessary for active muscle function during exercise. The current data does not support the use of quercetin for performance or resveratrol for recovery in warfighters.

Recovery from intense physical activity may also be significantly influenced by targeted nutritional supplementation, although often indirectly. Again, in data from sports athletes, protein consumed pre- and post-exercise provides the necessary substrates for muscle protein synthesis, aiding in the repair of muscle fibers that are damaged during exercise. This process is crucial for reducing delayed onset muscle soreness (DOMS) and promoting the adaptation required for future performance improvements [7]. Carbohydrates play a key role in replenishing glycogen stores that are depleted during exercise. Rapid glycogen resynthesis is critical for restoring muscle energy levels, reducing fatigue, and preparing the body for subsequent physical activity [7]. Polyphenols, particularly those found in beetroot juice, resveratrol, and quercetin, offer potent antioxidant and anti-inflammatory effects, as well as augment cytokine expression [31]. These compounds help neutralize reactive oxygen species generated during exercise, thereby reducing oxidative stress and muscle damage. The anti-inflammatory properties of polyphenols may also help mitigate DOMS and promote a faster return to training or competition [7]. Probiotics, though primarily associated with gut health, have been shown to influence muscle recovery by enhancing nutrient absorption and modulating systemic inflammation [32]. A healthy gut microbiome supports the efficient uptake of nutrients critical for muscle repair and can reduce systemic inflammation, further aiding in muscle recovery [32]. Together, these supplements create a comprehensive strategy to enhance both performance and recovery in physically demanding environments. In the current review, we examine whether these mechanisms also apply to warfighters or tactical athletes, as occupational demands and repeated performance requirements are quite unique compared to sports athletes and athletic competition (Figure 2).

### 4.2. Improvements in Physical Performance

During both periods of training and deployment, military personnel often confront nutritional challenges due to extreme calorie and nutrient deficits, limited access to fresh foods, and the repeated demands of field operations. Dietary supplementation can present an opportunity to ensure adequate intake of essential nutrients vital to optimize physical readiness and potentially reduce the risk of injury and fatigue. This parallels the use of supplements by sports athletes, highlighting the shared goal of maximizing performance and resilience in physically demanding contexts. However, there are unique differences between athletic and warfighter performance that require careful consideration, particularly with respect to fuel and nutrient intake. Supplementation strategies for sports athletes during controlled training and competition often prioritize performance optimization within a well-regulated environment. By contrast, supplementation for warfighters, both during training and field operations, places a premium on durability, adaptability, and sustained energy levels to endure prolonged periods of intense physical exertion and stress in unpredictable and resource-constrained conditions.

Findings on the benefits of protein and carbohydrate supplementation are somewhat diverse across study populations, although they have generally been demonstrated in sports athletes to improve muscle and sports performance [7]. Protein has been shown to improve performance indirectly by improving lean mass gains and, potentially, strength by providing a trigger for a rise in muscle protein synthesis and suppression of muscle protein breakdown [7]. Carbohydrate, on the other hand, provides a direct fuel source while maintaining blood sugar, thus improving both aerobic and anaerobic performance [7]. There is further evidence that insulin, secreted in response to carbohydrate intake, helps to improve muscle protein synthesis but, more importantly, lowers muscle protein breakdown post-exercise when adequate amino acids are available, highlighting additional indirect effects of carbohydrates when supplemented with proteins [28,33].

The results from the current review of warfighters, however, are limited and mixed regarding performance outcomes. Protein supplementation alone did seem to promote increases in strength in military personnel, as highlighted by McAdam et al. [20] and further evidenced by Walker et al. [25], who found that whey protein and leucine supplementation improved bench press and crunch performance in active-duty Air Force men [25]. However, it should be noted that these lab-based findings are clearly distinct from field studies, such as Berrymen et al. [12], who report that low-, moderate-, and high-protein diets post seven days of severely energy-restricted field training did not improve restoration of fat-free mass. However, field-based performance metrics were not assessed. This highlights the critical importance of studying supplementation in the military in a way that specifically addresses the unique job-related demands. Although protein supplementation can improve the physiological response to repeated exercise training, this does not provide insight into the effectiveness of protein supplementation in supporting performance and recovery in the field. Additional studies are needed to fully elucidate if protein and carbohydrate supplementation can be beneficial during field operations, and if so, what the appropriate recommendations are for protein supplementation during training compared to deployment. 

It appears that carbohydrate and protein supplementation, alone or in combination, may not be adequate to influence overall performance in tactical populations, although there are several factors outside of the supplementation protocol that may influence these results. It is important to consider dietary intake of protein and carbohydrates beyond supplementation patterns. As Berrymen et al. [12] suggested, if soldiers are consuming adequate amounts of carbohydrates and proteins through the diet, supplementation is not likely to influence muscle recovery. This is likely true for performance indicators as well. Despite evidence that timing of carbohydrate and protein intake may influence muscle performance and recovery, tactical populations are not consistently able to leverage these timing windows, suggesting that the overall amount of protein and carbohydrate intake in the diet is more important than following a supplemental regimen. This points to the importance of dietary behaviors in predicting physical performance and muscle recovery in military personnel. 

Single nutrient supplementation, particularly creatine and beta-alanine, has been shown to improve performance in sports athletes. Supplementation with 3–5 g/day of creatine has consistently demonstrated increases in muscle creatine stores, augmenting the rate of phosphocreatine resynthesis and enhancing short-term, high-intensity exercise capacity and the ability to perform repeated bouts of high-intensity events [7]. Beta-alanine at doses of ~65 mg/kg BM over a 10–12 week period provides an extracellular buffering effect in the blood, aiding intracellular pH regulation and leading to enhanced performance of short-term, high-intensity efforts [7].

The evidence on single nutrient supplementation was highly mixed for physical performance outcomes in warfighters and often contradicted the data noted in athletic populations. Seven days of creatine supplementation appeared to be effective at improving strength performance in military personnel when combined with beta-alanine [22], although when consumed as creatine alone, it showed no influence on strength as measured by pushups [11]. These findings are unique, based on the strength of evidence in support of creatine as a highly effective ergogenic aid in sports athletes [7] or other tactical athletes. Specifically, in firefighters, creatine added to a carbohydrate and protein supplement for approximately three weeks improved some occupational performance-related tasks, although not all [34]. It is likely that the dose and duration of creatine supplementation are key, and a loading regimen may be required to increase creatine storage and lead to a physiological effect [35]. In the studies reviewed, Samadi et al. [22] followed the loading regimen of 0.3 g/kg/day for seven days before measuring strength outcomes. Interestingly, Armentano et al. [11] supplemented with 20 g/day for seven days. This absolute dose is likely close to the recommended loading dose but may not produce the same storage effects as a relative dose would, based on the variety of soldier body composition. These findings may highlight the additional benefit of beta-alanine plus creatine on high-intensity exercise performance or may likely just call out the importance of identifying appropriate supplementation protocols to most effectively support improvements in performance. It is also important to understand what the supplementation protocol should look like after the loading phase to ensure adequate muscle performance support in field training exercises.

While beta-alanine may be effective at improving strength when combined with creatine, beta-alanine alone also has adequate evidence to suggest that it supports significant performance improvements in athletes [7]. Specifically, beta-alanine has been shown to have small but potentially meaningful performance benefits (~0.2–3%) during both continuous and intermittent exercise tasks of 30 s to 10 min in duration [36]. Hoffman et al. [16] also conducted a study on beta-alanine supplementation during field training and reported a therapeutic effect between military training events, potentially leading to better overall recovery and performance. These findings suggest that beta-alanine may be beneficial in performance and recovery in military personnel during both predictable and unpredictable settings. Unfortunately, the dose of beta-alanine in these two studies ranges from 6 g/day to 12 g/day, and the timing of supplementation is also variable, suggesting strong recommendations about beta-alanine supplementation cannot be made until more investigation can be completed. 

With regard to phytonutrient extracts, beetroot juice appeared to attenuate declines in fitness and heart rate recovery in soldiers after a field expedition [19]. Beetroot juice (i.e., dietary nitrate) enhances nitric oxide availability, which can result in increased blood flow to the working muscle, reduced ATP cost of muscle force production, and allows for increased efficiency of mitochondrial respiration [7]. Isolated nitrate supplementation, in particular, has demonstrated significant benefits for sports performance by improving time to exhaustion and high-intensity, intermittent activity lasting longer than 12 min [7]. This supplementation strategy may be worth additional investigation in the warfighter population due to the type of occupational activities required. Quercetin, however, did not improve physical performance metrics or perceived exertion during exercise testing in soldiers [23]. While the data on sports athletes are limited, a recent review does appear to demonstrate moderate efficacy for performance metrics, including aerobic capacity, time to exhaustion, and strength performance [37]. It is postulated that quercetin may have an anti-inflammatory, anti-oxidant effect while improving mitochondrial biogenesis, although these data have only limited support in humans [7].

Several studies examined nutrients in combination in the current review, with positive effects on performance and cognition. One study assessed the effectiveness of a mixed nutritional supplement for 12 weeks. Although the supplement did yield physical and cognitive performance benefits above placebo when controlling for an exercise regimen, the relevance of these findings is difficult to assess due to the proprietary blend of multiple nutrients [26]. It is further difficult to assess which ingredients likely influenced physical versus cognitive performance and whether these outcomes affected one another. Finally, the mixed supplement included a variety of macro- and micronutrients that could have influenced performance if those nutrients were being under-consumed in the diet. This once again highlights the importance of considering supplemental recommendations in the context of the daily diet in military settings. Although some multi-nutrient supplements may improve performance in the field, it is likely that others may not provide additional benefits above a well-balanced diet, suggesting the need for improvements in dietary behaviors rather than supplemental behaviors in the military.

### 4.3. Improvements in Recovery or Reduced Injury

The prevalence of injury in warfighters is unique from sports and athletic performance due to high occupational risk and distinctive long-duration demands. In a recent report, the total direct medical cost of treating MSIs among military trainees alone was approximately USD 15 million per year [38]. However, dietary supplementation has been shown to attenuate injury burden among sports athletes [7], and this may also be true for military populations. In response to this emerging recognition of significant human and economic burden, a recent report by the Department of Defense published in March 2023 concluded that the Military Health System must “reshape its focus on disease and injury treatment and prevention to embrace health enhancement for optimal human performance in a technology-rich battle space” [39].

Evidence from the current systematic review demonstrates that supplementation with additional calories from protein and carbohydrate [12], whey protein alone [20], probiotics [14,17], beta-alanine [16], and oregano [24] may be promising for reducing inflammation and markers of muscle damage, thus, plausibly reducing injury and enhancing recovery. Conversely, the polyphenol resveratrol [18] did not demonstrate any effect on oxidative stress or inflammatory biomarkers. Only one study specifically measured decreased injury from supplementation in the tactical population [21], and none of the studies demonstrated direct improvements in recovery specifically.

Protein, carbohydrate, and overall adequate calorie intake have been shown to improve recovery and reduce the risk of injury [7] and complications associated with relative energy deficiency in sport (RED-S) [40] in sports athletes. Further, some specific amino acids, such as glutamine, are an important energy substrate for immune cells, and circulating glutamine is lower after prolonged exercise and heavy training [7]. Collagen proteins have also been shown to thicken cartilage and decrease joint pain after injury, although evidence is quite limited on recovery in sports athletes [7]. Exogenous carbohydrates have been demonstrated to lower stress hormones and counter immune dysfunction at doses of 30–60 g/h, although very limited evidence demonstrates any mitigation of infection risk [7]. With regard to overall calorie and macronutrient supplementation on markers of recovery and injury in the tactical population, the data retrieved were limited, and effect sizes were small. Berryman et al. [12] demonstrated that in the presence of adequate protein, restoration of caloric intake rather than higher levels of protein was responsible for driving FFM recovery after short-term severe negative energy balance. This conclusion was mirrored in soldiers participating in Army Initial Entry Training, where whey protein supplementation resulted in significant fat mass loss but did not affect FFM [20], as well as in Air Force airmen who demonstrated no effect of whey protein plus leucine supplementation on FFM [25]. In the only study that directly measured injury, McGinnis et al. [21] demonstrated a direct reduction in MSI outcomes in a population of soldiers participating in Army Initial Entry Training with whey protein or carbohydrate supplementation, although this study was limited due to historical comparisons rather than a dedicated control. 

Overall, there are limited data on the utility of probiotic supplementation for recovery or injury prevention, although there are early associations with improved body composition and lean body mass, hormonal response to training, reduction of exercise-induced lactate, and mental health in sports athletes [41]. It is postulated that the polysaccharides derived from the cell walls of the microbiota can stimulate innate immunity, although this mechanism has yet to be demonstrated in humans [7]. In the current review, two studies specifically examined probiotic supplementation in tactical athletes [14,17]. Gepnar et al. [14] demonstrated that with the consumption of probiotics, bacillus coagulans, there is a potential increase in muscle integrity and a decrease in cytokine response after demanding military training. Meanwhile, a second study examining bacillus coagulans demonstrated no changes in blood biomarkers of muscle damage after a physical training test, including cytokines [17]. Neither study reported specifically on outcomes of recovery or injury prevention.

The utilization of beta-alanine has been shown to improve performance outcomes in sports athletes [7], but evidence examining recovery or injury prevention is scarce. In the singular study in soldiers participating in intense military training, beta-alanine appeared to non-significantly increase circulating IL-10 [16], an anti-inflammatory cytokine associated with performance and resilience in members of the military [42]. However, this study was impaired with significant attrition (50%) in the supplementation group, and the findings are difficult to interpret.

Polyphenol and herbal supplements are often used for recovery, muscle soreness, and injury management by sports athletes, although the data are highly variable and limited [7]. Plant flavonoids, in particular, have been demonstrated, in vitro, to have strong anti-inflammatory, anti-oxidant, and anti-pathogenic effects [7]. In the tactical population, only two studies [18,24] on polyphenols and herbals were reported with regard to recovery and injury, with minimal effect. Shirvani and colleagues [24] demonstrated that oregano (organum vulgare) can decrease markers of muscle damage creatine kinase, malondialdehyde, and lactate dehydrogenase levels after the army combat readiness test. Conversely, when military firefighters were exposed to resveratrol daily for three months, there was no effect on plasma oxidative stress markers or cytokines after a typical fitness test [18]. However, no injury or recovery measures were reported post-supplementation in either study. 

### 4.4. Limitations

The major limitation of this systematic review was the scarcity of controlled trials in the literature and the lack of consistency between ingredient, dose, population, and study design, including specific outcomes related to muscle quality, performance, recovery, or injury. An existing body of evidence is particularly lacking in the context of female military populations, making generalizability of results difficult between sexes. This is also true in the sports supplementation literature [43]. It is further important to note that few of these studies controlled for the military diet in any way, and supplementation effects are likely related to diet quality. There is also a lack of control for other behaviors like sleep and overall diet quality in the included studies, which has been identified as problematic in the warfighter population{Citation}. It is difficult to draw conclusions on the influence of supplements on physical performance and recovery without also knowing the overall quality of both diet and sleep [44]. Finally, there is a significant lack of field-based testing relevant to warfighters during lengths of active-duty deployment. The existing lab-based findings are promising, but there is a critical need for further investigation to ascertain whether outcomes affect warfighters in real-world field environments. The complexity and unique demands of military operations necessitate a robust and nuanced understanding, which can only be achieved through a more extensive and targeted research effort.

## 5. Conclusions

This systematic review sought to outline the demonstrated impact of dietary supplements on muscle-related physical performance and recovery outcomes in the warfighter population. The available evidence suggests some, although not all, dietary supplements may modestly improve physical performance in this population. Even fewer supplements appeared to reduce inflammation and, thus, improve recovery in warfighters. Protein and carbohydrate supplementation had a positive effect on physical performance and helped to reduce inflammation, especially when there is limited access to a well-balanced diet. B-alanine also appeared to improve physical performance and reduce markers of inflammation and may further improve performance when combined with creatine. Beetroot juice benefited high-intensity, moderate-duration performance. Probiotics and oregano helped to reduce markers of inflammation, while resveratrol did not. No harmful outcomes were identified in the studies reviewed. Further interventional research, particularly in the field setting, is required to understand optimal supplemental protocols and practical differences in supplemental support during military training versus active-duty assignments. The results of this systematic review highlight the unique challenges faced by military personnel and call for continued exploration of nutritional interventions to enhance overall resilience and physical capabilities in the complex landscape of military operations.

## Figures and Tables

**Table 1:** Database and Search Term Strategy

Database	Search Terms Used
CINAHL	(Military personnel or soldiers or armed forces or service men) AND (supplements dietary) OR strength OR performance OR recovery OR muscle OR strength performance AND (“randomized controlled trials”) AND (“longitudinal”)—peer reviewed, randomized control trials, past 10 years, 19–44 years old
Medline	(((supplement OR protein OR “vitamin D” OR “fish oils” OR “omega 3” OR “collagen” OR “creatine” OR “beta alanine”)) AND (“tactical athlete” OR soldier OR warfighter OR sailor OR army OR marine OR navy OR coast guard OR air force)) AND (“brain health” OR “mental health” OR “performance” OR “recovery” OR “strength”)

**Table 2:** Participants, Interventions, Comparisons, Outcomes, and Study Design Criteria.

Participants	Active-duty military members, aged 19 years or older
Intervention	Dietary supplementation defined as the provision of nutrients or food separate from the diet OR as a part of the diet AND approved by Department of Defense
Comparisons	Control group receiving a placebo or no nutrient/dietary supplementation
Outcomes	Subjective and objective measures of muscle performance, recovery, or body composition
Study Design	Randomized Controlled Trial

**Table 3:** Characteristics of Included Studies.

Reference	Participants	Study Design	Nutrition Intervention	Primary Physical Outcomes	Results
Armentano et al., 2017 [11]	United States Army soldiers, n = 20 men and n = 15 women	Double-blind, placebo controlled	Group 1: 5 g creatine, 4× per day; Group 2: 5 g taurine, 16 g sucrose, 4× per day for 7 days	Performance	2 min pushup: no significant difference in creatine and taurine groups (*p* = 0.0437) Creatine: serum creatine levels (*p* < 0.001), no changes in body composition, weight, blood pressure or serum phosphokinase levels
Berryman et al., 2017 [12]	United States Marines, n = 63 male	Randomized, double-blind, placebo controlled	Group 1: Higher protein (133 g/day); Group 2: Moderate protein (84 g/day); Group 3: Carbohydrate based low protein (7 g/day); Intervention was 27-days after 7 days of caloric restriction (CR) during training	Recovery	Total body mass (TBM; 5.8 1.0 kg, 7.0%), FFM (3.1 1.6 kg, 4.7%), and net protein balance (1.7 1.1 g protein·kg 1 ·day 1) were lower after CR. After 27-days, TBM (5.9 1.7 kg, 7.8%) and FFM (3.6 1.8 kg, 5.7%) improved in all groups.
Fortes et al., 2011 [13]	British Soldiers, n = 30 male	Quasi-Experimental Controlled	Group 1: habitual diet alone (CON); Group 2: habitual diet and daily mixed supplement with increased calories (SUP); Intervention was 8 weeks	Performance	Body mass loss (mean ± SD) (CON 5.0 ± 2.3, SUP 1.6 ± 1.5 kg), lean mass loss (CON 2.0 ± 1.5, SUP 0.7 ± 1.5 kg), and fat mass loss (CON 3.0 ± 1.6, SUP 0.9 ± 1.8 kg) were significantly blunted by SUP. CON experienced significant decrements in maximum dynamic lift strength (14%), vertical jump (10%), and explosive leg power (11%) that were prevented by SUP.
Gepner et al., 2017 [14]	Israel Defense Force soldiers; n = 26 male	Double-blind, parallel design	Group 1: Beta hydroxy beta methylbutyrate calcium (CaHMB; 3 g) with Bacillus coagulans (BC30); Group 2: CaHMB 3 g with placebo; Group 3: control group; Intervention was 20 days.	Recovery	Significant attenuation in IL-1, IL-2, IL-6, CX3CL1, and TNF for both CaHMBBC30 and CaHMBPL compared with the control. Significant differences post test in plasma IL-6 (F = 6.27, *p* = 0.012) and IL-10 (F = 3.72, *p* = 0.041) concentrations. Positive effects on muscle integrity in rectus femoris (*p* = 0.014) and vastus lateralis (*p* = 0.23). No changes in body mass between groups (*p* = 0.22).
Hoffman et. al., 2015 [15]	Israel Defense Force Soldiers; n = 18 male	Randomized, double-blind study, placebo controlled	Group 1: 6 g/day beta-alanine; Group 2: 6 g/day placebo (rice flour); Intervention was 30 days	Recovery	Elevation in muscle carnosine content (*p* = 0.048) associated with improvement in fatigue (*p* = 0.06). Improvement in 50 m casualty carry (*p* = 0.044), and serial subtraction test (*p* = 0.022) with no differences in 1 min sprint, repeat sprint, marksmanship performance, or 2.5 km run.
Hoffman et. al., 2018 [16]	Israel Defense Force Soldiers; n = 20 male	Double-blind, parallel field study	Group 1: 6 g/day of sustained release beta-alanine (BA); Group 2: 6 g/day of rice powder (PL); Intervention was 30 days	Recovery	Changes in circulating IL-10 concentrations (mean difference 0.86 pg/mL) was possibly greater (57%) for BA than PL
Hoffman et. al., 2018 [17]	Israel Defense Force Soldiers; n = 16 male	Double-blind, parallel design	Group 1: Inactivated Bacillus coagulans (iBC) supplement, 1.0 × 10^9^ colony-forming units; Group 2: Placebo; Intervention was 14 days.	Performance	In all analyses of performance (jump test, maximum pull ups, simulated casualty drag, shuttle run) and blood (cytokines and chemokines), there were no significant differences between groups. Magnitude based inferential analysis demonstrated changes in jump test and the casualty drag could have been advantageous (90.7% and 80.4% likelihood effect)
Macedo et. al., 2014 [18]	Brazilian military firefighters, n = 60 male	Triple-blind, placebo controlled	Group 1: 100 mg of resveratrol; Group 2: 100 mg of placebo; Intervention was 90 days	Recovery	Glucose (*p* = 96.06 ± 2.67) and triglycerides (*p* = 5.4 ± 1.43) were elevated post test in resveratrol group, but IL-6 and TNFa were reduced by 29% and 39%. Total antioxidant capacity was increased in both resveratrol and placebo groups.
Marshall et. al., 2021 [19]	British Military Soldiers; n = 12 male, n = 10 female	Single-blind, randomized control	Group 1: 14-mL concentrated beet root juice (BRJ; 12.5 mmol nitrate); Group 2: 14 mL calorie, color, volume—matched dose with 15.4 g maltodextrin, 2.8 g protein, 14 mL’s blackcurrant cordial with negligible phytochemical content and 70 mL’s Buxton; Intervention was 17 days.	Performance	BRJ enhanced the salivary levels of nitrite (*p* = 0.007). Harvard Step Test: Scores declined as altitude increased in control group (*p* = 0.003), no decline as altitude increased (*p* = 0.26) in BRJ group. Heart rate recovery was prolonged in control group (*p* < 0.01), unchanged with BRJ (*p* = 0.61)
McAdam et al., 2017 [20]	United States Army Soldiers, n = 69 male	Repeated measure, double-blind, parallel group	Group 1: Whey protein (77 g/day), 2× per day; Group 2: Energy matched carbohydrate (127 g/day, 2× per day; Intervention was 8 weeks	Performance	Post-testing pushup averaged 7 repetitions more in whey protein group (*p* < 0.001) with post-training fat mass loss (*p* = 0.01). No change in run time (*p* = 0.065) or fat free mass (F = 0.70, *p* = 0.41)
McGinnis et. al., 2018 [21]	United States Army Soldiers, n = 2175 male	Quasi experimental, double-blind, controlled	Group 1: One protein (38.6 g, 293 kcal), after physical training and before bed, or before bed only; Group 2: One carbohydrate (63.4 g, 291 kcal), after physical training and before bed, or before bed only; Group 3: Two protein (77.2 g, 586 kcal), after physical training and before bed, or before bed only; Group 4: Two carbohydrate servings/day (126.8 g, 582 kcal) after physical training and before bed, or before bed only; Control: historical data	Injury	Non supplemented soldiers were 5× more likely to sustain musculoskeletal injury (MSI) (*p* < 0.001) and 4× more likely to miss training *p* = 0.003) compared totwoservings of protein; Non supplemented soldiers missed 5 additional training days (*p* = 0.02) compared totwoservings of protein. One serving of protein per day was 3× more likely to have MSI thantwoservings (*p* = 0.002)
Samadi et. al., 2022 [22]	Iranian Soldiers, n = 20 male	Double-blind, randomized	Group 1: 6.4 g/day beta-alanine plus 0.3 g/kg creatine (BA + Cr); Group 2: 6.4 g/day beta-alanine plus placebo (BA + Pl); Intervention was 28 days.	Performance	BA + Cr increased bench press (*p* = 0.026), leg press (*p* = 0.017), vertical jump (*p* = 0.009), and sprint power (0.023) and simulated casualty test (*p* = 0.003), while BA + Pl did not. Vertical jump (*p* = 0.005) and testosterone (*p* = 0.006) were increased in BA + Cr compared to BA + Pl. No changes in cortisol, IGF-1, or lactate in either group.
Sharp et. al., 2012 [23]	United States Army Soldiers, n = 16 male	Double-blind, placebo controlled, crossover study	Group 1: 1000 mg of quercetin (food bar); Group 2: 1000 mg of placebo (food bar); Intervention was 7 days.	Performance	Quercetin supplementation did not show any aerobic changes in performance (VO2peak, time trial, respiratory exchange ratio, ratings of perceived exertion).
Shirvani et al., 2022 [24]	Iranian Soldiers, n = 24 male	Randomized block design	Group 1: 500 mg oregano immediately after exercise; Group 2: 500 mg of placebo (starch); Intervention only measured after one day of supplementation and testing.	Recovery	Oregano supplementation demonstrated improvements in creatine kinase (*p* < 0.0001), lactate dehydrogenase (*p* < 0.0001), malondialdehyde (*p* < 0.0001), super oxide dismutase (*p* < 0.0001), antioxidant capacity (*p* < 0.0001) and glutathione peroxidase (*p* < 0.0001). In all variables, the difference between placebo and oregano groups were significant at 60 (*p* < 0.0001) and 120 (*p* < 0.0001) minutes after army combat readiness test.
Walker et al., 2010 [25]	United States Air Force Airmen, n = 24 male, n = 6 male non-military	Randomized, double-blind	Group 1: Whey protein (19.7 g) and leucine (6.2 g); Group 2: Energy matched placebo; Intervention was 8 weeks	Performance	Overall, 55.6% of participants consuming whey protein demonstrated 5% or greater improvement compared to only 16.7% of individuals in the placebo group (*p* = 0.033). Whey Protein saw improvement of 12.8% pushup and 7.2% crunch increase while, placebo showed 7.6% and 3.4% increases, respectively with no between group differences. No difference was noted for run time, pull ups, or cognitive tests.
Zwilling et al., 2020 [26]	United States Airforce Airmen, n = 107 male and n = 41 female	Randomized Controlled Trial	Group 1: Two 8-oz mixed nutritional supplement (Protein, Carbohydrate, Fat, Ca-HMB, Choline, DHA, Folic Acid, Lutein, Magnesium, Phospholipid, Selenium, Vitamins: B1, B2, B3, B5, B6, B12, C, D, E, and Zinc) plus exercise; Group 2: Two 8-oz placebo plus exercise; Intervention: 12 weeks.	Performance	Relative to exercise alone, the mixed ingredient supplement resulted in increased lean muscle mass (*p* = 0.0003), decreased heart rate (*p* = 0.007), improved working memory (*p* = 0.01) and processing efficiency (*p* = 0.08). There was no significant difference between placebo and supplementation for strength and endurance, mobility and stability, or power.
